# Manganese boosts natural killer cell function via cGAS–STING mediated UTX expression

**DOI:** 10.1002/mco2.683

**Published:** 2024-08-28

**Authors:** Qianyi Ming, Jiejie Liu, Zijian Lv, Tiance Wang, Runjia Fan, Yan Zhang, Meixia Chen, Yingli Sun, Weidong Han, Qian Mei

**Affiliations:** ^1^ Department of Bio‐Therapeutic the First Medical Center Chinese PLA General Hospital Beijing China; ^2^ Central Laboratory National Cancer Center/National Clinical Research Center for Cancer/Cancer Hospital & Shenzhen Hospital Chinese Academic of Medical Sciences and Peking Union Medical College Shenzhen China; ^3^ Changping Laboratory Beijing China

**Keywords:** antitumor immunity, cGAS–STING, manganese, natural killer cells, UTX

## Abstract

Natural killer (NK) cells play a crucial role in both innate immunity and the activation of adaptive immunity. The activating effect of Mn^2+^ on cyclic GMP‐AMP(cGAS)–stimulator of interferon genes (STING signaling has been well known, but its effect on NK cells remains elusive. In this study, we identified the vital role of manganese (Mn^2+^) in NK cell activation. Mn^2+^ directly boosts cytotoxicity of NK cells and promotes the cytokine secretion by NK cells, thereby activating CD8+ T cells and enhancing their antitumor activity. Furthermore, Mn^2+^ can simultaneously activate NK‐cell intrinsic cGAS and STING and consequently augment the expression of ubiquitously transcribed tetratricopeptide repeat on chromosome X (UTX to promote the responsiveness of NK cells. Our results contribute to a broader comprehension of how cGAS–STING regulates NK cells. As a potent agonist of cGAS–STING, Mn^2+^ provides a promising option for NK cell‐based immunotherapy of cancers.

## Graphical abstract

Schematic illustration of manganese promoting the activation and function of NK cells in tumor tissues. Manganese increased constitutive activation of cGAS and subsequently stimulated the STING/interferon regulatory factor 3 (IRF3)/Type‐I interferon (IFN‐I) pathway, promoting expression of UTX. Therefore, NK cells exhibited enhanced killing and secreting activity, and played a critical antitumor role in synergy with T cells.

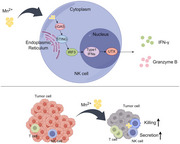

## 1 INTRODUCTION

In recent decades, cancer treatment has shifted toward using immune checkpoint blockers, adoptive immune cell infusion, and other tumor immunotherapies as the primary options. These treatment strategies primarily harness the endogenous cytotoxicity of CD8+ T cells to destroy cancer cells, this frequently leads to the development of drug resistance in the cancer cells, hence diminishing the treatment's effectiveness. The identification of innate immune sensing as a crucial trigger for innate and adaptive antitumor immune responses has been established in both natural and therapeutic biological scenarios.^1^ Natural killer (NK) cells are a type of lymphocytes that belong to the innate immune system and be regarded as the innate counterparts of CD8+ cytotoxic T cells.^2^

NK cells are “first responders,” possessing the ability to rapidly identify and eliminate infected, transformed, allogeneic, or stressed cells without prior encounter.^3^, ^4^, ^5^, ^6^, ^7^, ^8^, ^9^ They function as both direct and supporting contributors in antitumor immune responses by swiftly eradicating cancer cells through targeted cytotoxicity mechanisms and effectively producing proinflammatory cytokines and chemokines that recruit and activate other immune cells to generate an adaptive response.^10^, ^11^, ^12^, ^13^ NK cells possess these characteristics that allow them to enhance adaptive immune responses against malignancies and directly eliminate cancers that have evaded T cell responses. This makes NK cells very promising candidates for immunotherapy. The cyclic GMP‐AMP (cGAS)–stimulator of interferon genes (STING) cascade is crucial in the innate immune system's detection of cytoplasmic DNA^14^, ^15^, ^16^ and in promoting the cancer‐immunity cycle. The activation of this pathway results in the production of type‐I interferons (IFN‐I) (IFNα and IFNβ),^17^ as well as proinflammatory cytokines and chemokines.^18^, ^19^, ^20^, ^21^, ^22^, ^23^, ^24^ 2′,3′‐cyclic GMP–AMP (cGAMP) is produced when double‐stranded DNA (dsDNA) is cleaved by cGAS, which then binds to STING to recruit protein kinase TANK binding kinase 1 (TBK1).^18^, ^25^, ^26^, ^27^, ^28^, ^29^ The cGAMP–STING complex activates the transcription factors interferon regulatory factor 3 (IRF3) and nuclear factor‐κB (NF‐κB), which in turn create type‐I IFN, IFN‐responsive genes, and other cytokines and chemokines.^30^, ^31^ Extrinsic cGAS–STING activation of NK cells has been previously described in tumor models. Tumor‐derived second messenger cGAMP can activate NK cells’ endogenous STING. It can also activate STING in nearby myeloid cells, which in turn causes type I IFN production and increases NK cells’ antitumor activity. Nevertheless, the precise mechanism by which NK cell–intrinsic cGAS signaling and its downstream critical genes regulate NK cell activity remains unclear.

Furthermore, intratumoral administration of STING agonists, such as cGAMP, has demonstrated the enhancement of the antitumor activity of NK cells through their effects on both NK cells and myeloid cells. Nevertheless, the feasibility of intratumoral delivery is restricted. Manganese (Mn^2+^) can directly activate cGAS, raise cGAS's sensitivity to double‐stranded DNA (dsDNA, and increase STING's affinity for binding to cGAMP. As a result, Mn^2+^ can function as a potent agonist of the cGAS–STING cascade at multiple levels, thereby enhancing INF‐I production.^32^, ^33^, ^34^ Notably, as an essential metal element, Mn^2+^ has been extensively studied for its toxicity and can be safely administered systemically.^35^, ^36^, ^37^, ^38^ Given its easy availability and extensively researched toxicity, Mn^2+^ could be an attractive option to augment NK cell‐based cancer immunotherapy as it acts as a pan‐agonist of cGAS and STING.

In this study, we show that the intrinsic cGAS signaling enhances the antitumor ability of NK cells after Mn^2+^ administration, independent of cGAMP from tumor cells. Moreover, Mn^2+^ can improve the efficacy of NK cells in eliminating cancer cells and influence the secretion profile of NK cells, leading to boosted activation of T cells and remodeling of the tumor microenvironment. Mechanistically, Mn^2+^ facilitates the activation of NK cells by inducing the production of ubiquitously transcribed tetratricopeptide repeat on chromosome X (UTX), a crucial molecular mediator involved in the effector responses of NK cells, through the cGAS–STING pathway. Hence, our results propose alternate strategies for enhancing NK cell‐based cancer therapies.

## 2 RESULTS

### 2.1 Antitumor effect of Mn^2+^ depends on NK cells

**FIGURE 1:**
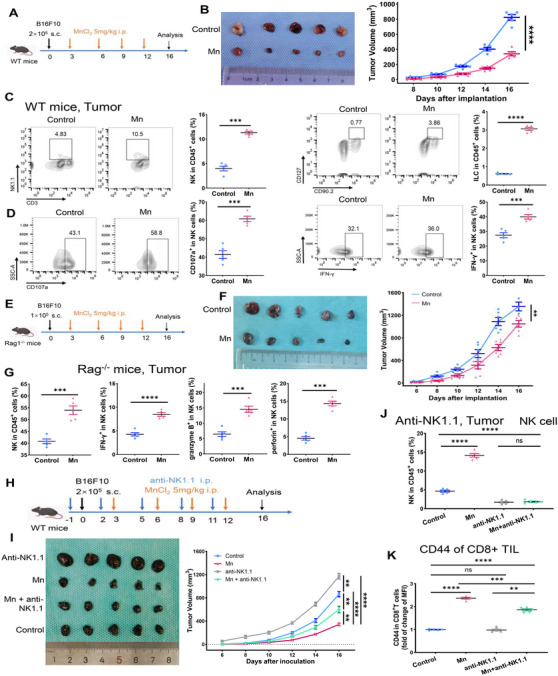
NK cells mediate the antitumor effects of Mn^2+^. (A) 2 × 10^5^ B16F10 cells were inoculated subcutaneously to the wild‐type mice with or without 5 mg/kg MnCl_2_ intraperitoneal treatment. (B) Representative images (left) and tumor growth curve (right) of B16F10 tumor in control and Mn^2+^‐treated WT mice (*n* = 5 per group). (C) Representative FACS data and statistics of frequency of tumor infiltrating NK cells (left) or ILCs (right) of mice as in (B) (*n* = 5 per group). (C) Representative FACS data and quantification of the frequency of tumor infiltrating CD107a+ NK (left) or IFNγ+ NK cells of mice as in (B) (*n* = 5 per group). (E) 1 × 10^5^ B16F10 cells were implanted subcutaneously to the Rag^−/−^ mice with or without intraperitoneal injection of 5 mg/kg MnCl_2_. (F) Images of tumors (left) and quantification of tumor sizes (right) in B16F10 tumor in Rag^−/−^ mice treated with or without MnCl_2_ (*n* = 5 per group). (G) As mice in (F), the frequency of tumor infiltrating NK cells (left), and IFNγ+, granzyme B+, and perforin+ NK cells (right) were quantified by flow cytometry (*n* = 5 per group). (H) NK cells were depleted from WT mice by delivering anti‐NK1.1 antibody every 3 days. 2 × 10^5^ B16F10 cells were inoculated to the NK cell‐depleted mice with or without 5 mg/kg MnCl_2_ intraperitoneal administration. (I) Representative images (left) and tumor growth curve (right) of B16F10 tumor in control and Mn^2+^‐treated WT or NK cell‐depleted mice (*n* = 5 per group). (J and K) Frequency of tumor infiltrating NK cells (upper) and expression of CD44 on CD8+ TILs (lower) of mice as in (I) were quantified by flow cytometry (*n* = 5 per group). Data represent analyses of the indicated *n* mice per group, means ± SEM. ^**^ *p* < 0.01; ^***^ *p* < 0.001; ^****^ *p* < 0.0001; ns, not significant, *p* > 0.05.

To verify the antitumor effect of systemic administration of Mn^2+^, we established tumors by injecting murine melanoma cells B16F10 subcutaneously in C57/BL6 mice. The mice were then treated with MnCl_2_ intraperitoneally on four times (Figure 1A). Administration of Mn^2+^ severely impaired tumor growth (Figure 1B). Compared with the control tumors, the increased accumulation of NK cells, innate lymphoid cells (ILCs), CD8+ T cells, CD4+ T cells, dendritic cells (DCs), and natural killer T cells (NKT cells) were observed within the Mn^2+^‐treated tumors (Figures 1C and S1A,B). Furthermore, we demonstrated enhanced antitumor activity of NK cells characterized by CD107a, Interferon γ (IFNγ), granzyme B (GZM), and perforin within the Mn^2+^‐treated tumors (Figures 1D and S2A). Consistent with the effects of systemic administration, Mn^2+^ injection also increased the concentration of the aforementioned immune cells and NK cell activation in the peripheral blood and spleen (Figures S1C–F and S2B–D).

To gain a deeper understanding of the primary factors that contribute to the anticancer effect of Mn^2+^, we conducted experiments using Rag1^−/−^ mice, which are deficient in T and B cells. This allowed us to eliminate the influence of T or B cells (Figure 1E). The anticancer effects of Mn^2+^ were partly diminished but remaining significant (Figure 1F). The treatment also induced robust activation of NK cells in the tumor, peripheral blood, and spleen (Figures 1G and S1G,H). The observations indicated that NK cells play a crucial role in the antitumor effect caused by the Mn^2+^ injection.

To further explore the role of NK cells in Mn^2+^ antitumor effect, we established a mouse tumor model with antibody‐mediated depletion of NK cells (Figure 1H). NK cell depletion led to accelerated tumor growth and significantly attenuated the antitumor impact of Mn^2+^ (Figure 1I,J). Nevertheless, the growth of the tumor was only partially suppressed, since there was still some level of activation of CD8+ T cells observed following treatment with Mn^2+^ (Figure 1I,K). Collectively, these results indicate that Mn^2+^ mostly affects NK cells directly and may also modulate other immune cells either by activating NK cells or independently of NK cell activation.

### 2.2 Mn^2+^ directly mediated the cytotoxic effect of NK cells

**FIGURE 2:**
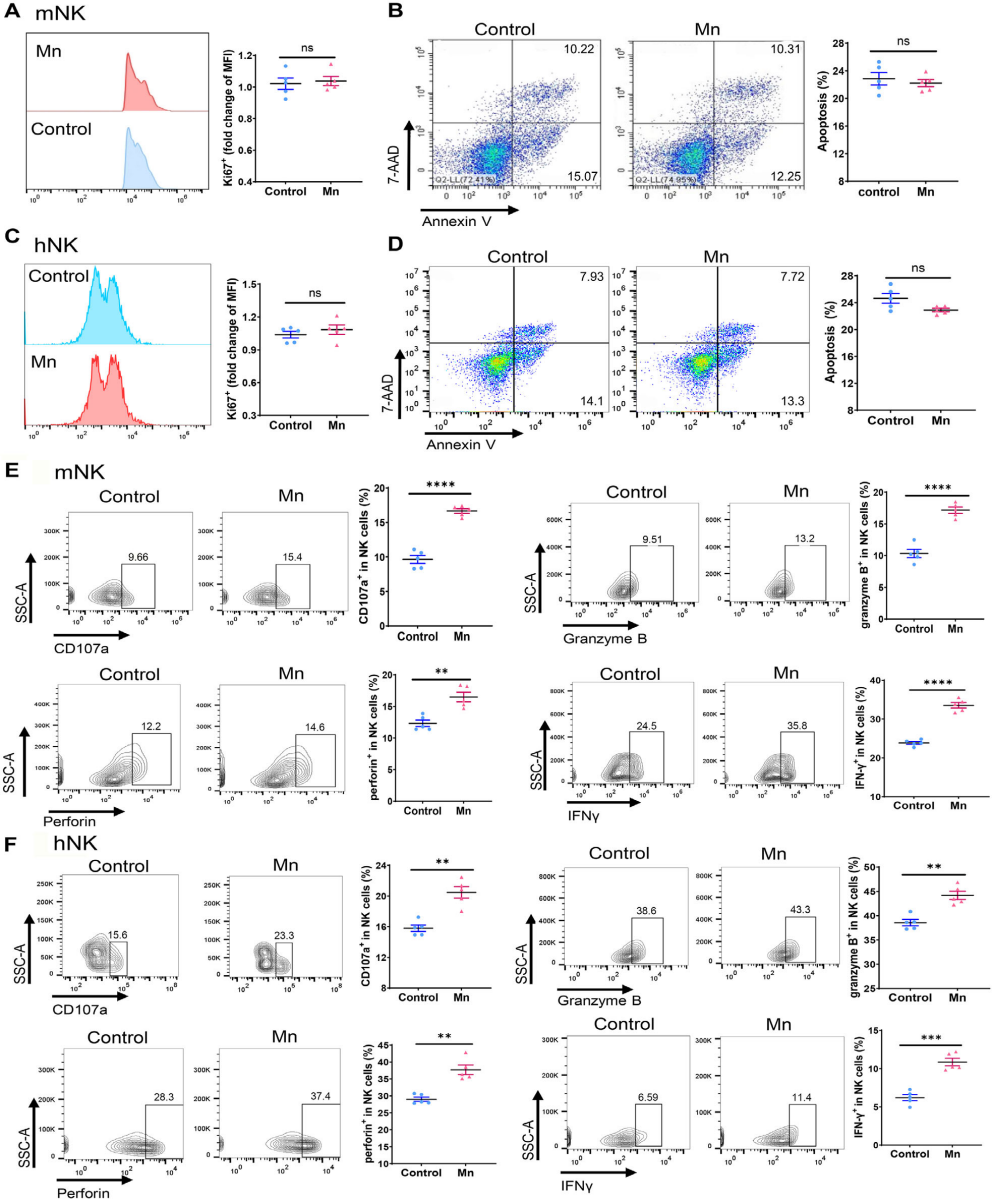
Mn^2+^ enhances the activation of NK cells. (A–D) Primary murine and human NK cells were isolated from spleens of mice and PBMCs of humans, respectively, and then preactivated by IL‐2 and IL‐15. Preactivated murine NK cells were incubated with or without Mn^2+^ for 24 h in vitro. Apoptosis (A and C) and proliferation (B and D) of treated NK cells were evaluated by flow cytometry (left) and subjected to statistical analysis (right) (*n* = 5 per group). (C) As murine NK cells cultured in (A), the effect factors of murine NK cells, namely CD107a, IFNγ, granzyme B, and perforin, were assessed by flow cytometry (left) and performed statistical analysis (right) (*n* = 5 per group). (D) As ex vivo human NK cells in (B), Frequency of CD107a+, IFNγ+, granzyme B+, and perforin+ human NK cells were analyzed by flow cytometry (*n* = 5 per group). Data represent analyses of the indicated *n* mice per group, means ± SEM. ^**^ *p* < 0.01; ^***^ *p* < 0.001; ^****^ *p* < 0.0001; ns, not significant, *p* > 0.05.

NK cells have a vital function in identifying and eliminating tumor cells. Our next objective was to explore the potential role of Mn^2+^ in the cytotoxicity of NK cells. We employed NK cells purified from murine spleens and human peripheral blood mononuclear cells (PBMCs) to validate the impact of Mn^2+^ on NK cell activation ex vivo. Mn^2+^ did not have a notable impact on the growth and apoptosis of both human and mouse NK cells, indicating that the enhanced antitumor effect of NK cells is a result of direct activation (Figure 2A–D). Exposure to Mn^2+^ could enhance the expression of CD107a and stimulate the production of IFNγ, granzyme B, and perforin in both murine and human NK cells in vitro (Figure 2E,F).

**FIGURE 3:**
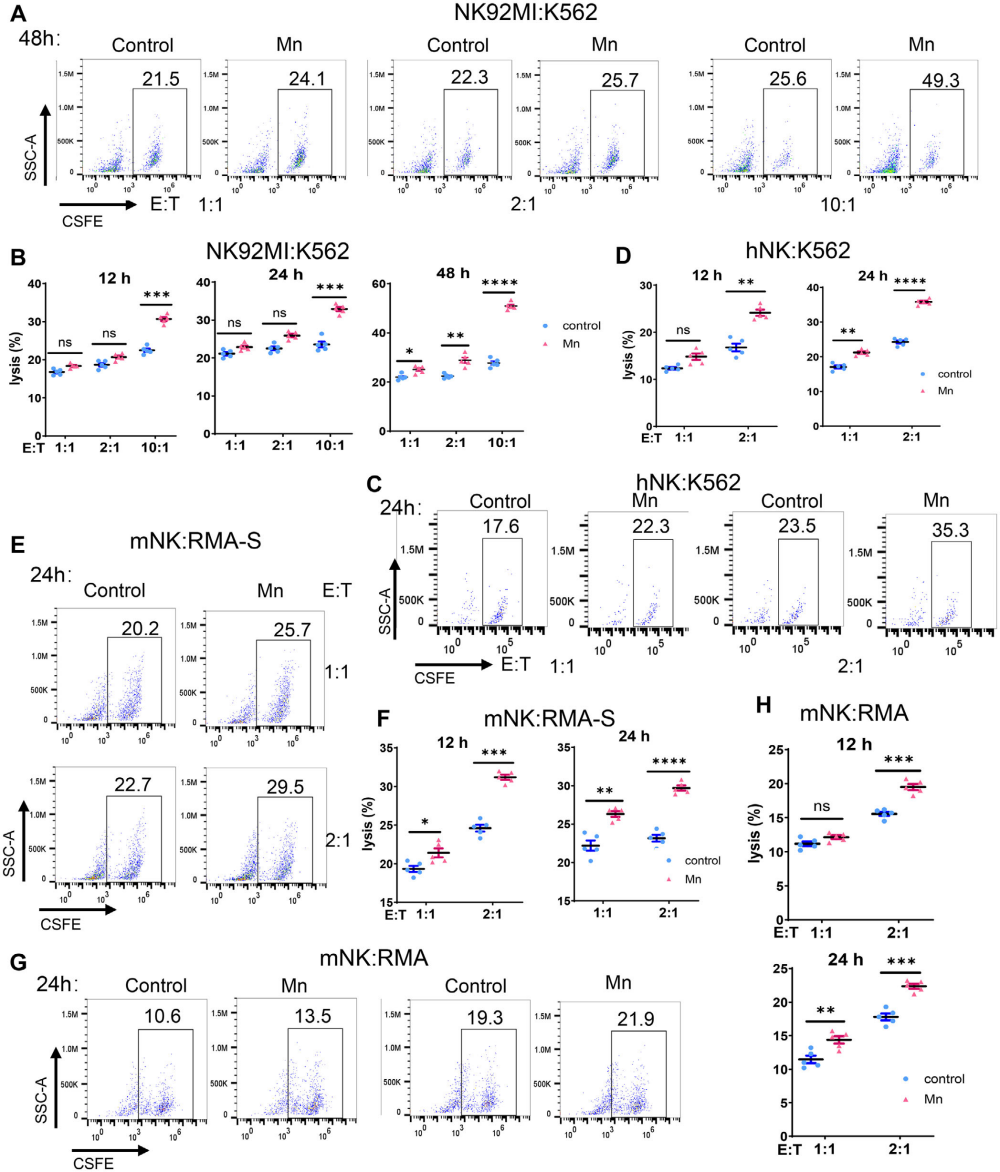
Mn^2+^ directly improves the cytotoxicity of NK cells against tumors. (A–D) NK92‐MI cell line or preactivated primary human NK cells were cocultured with target cell K562 at various effector–target ratios, in the presence or absence of Mn^2+^. The killing ability of NK cells against K562 cells were evaluated by flow cytometry (A and C) (*n* = 5 per group). The lysis frequencies of K562 cells were statistically analyzed (B and D) (*n* = 5 per group). (E–H) Primary murine NK cells were preactivated and coculture with target lymphoma cell TAP2‐deficient RMA‐S or wild‐type RMA cells with or without Mn^2+^. Representative FACS data (E and G) and statistics (F and G) of frequency represent the tumor killing ability of murine NK cells (*n* = 5 per group). Data represent analyses of the indicated *n* mice per group, means ± SEM. ^*^ *p* < 0.05; ^**^ *p* < 0.01; ^***^ *p* < 0.001; ^****^ *p* < 0.0001; ns, not significant, *p* > 0.05.

We further conducted analysis on the impact of Mn^2+^ on the cytotoxicity of NK cells by utilizing an in vitro coculture system of NK cells and target cells. Prolonged exposure to Mn^2+^ and enhanced effect‐target ratio led to a considerable increase in the cytotoxicity against tumor cells in vitro by both the NK‐92MI cell line and human primary NK cells (Figures 3A–D and S3A,B). The results clearly demonstrated a consistent and noticeable improvement in the killing capacity of primary murine NK cells against TPA2‐deficient RMA‐S lymphoma cells. Meanwhile, the elevated cytotoxicity of NK cells is noteworthy in wild‐type RMS cells, although being somewhat abolished (Figures 3E–H and S3C,D). To summarize, Mn^2+^ enhances the anticancer effect of NK cells by directly increasing their ability to kill tumors.

### 2.3 Mn^2+^ boosts the activation of CD8+ T cells by functioning on NK cells

**FIGURE 4:**
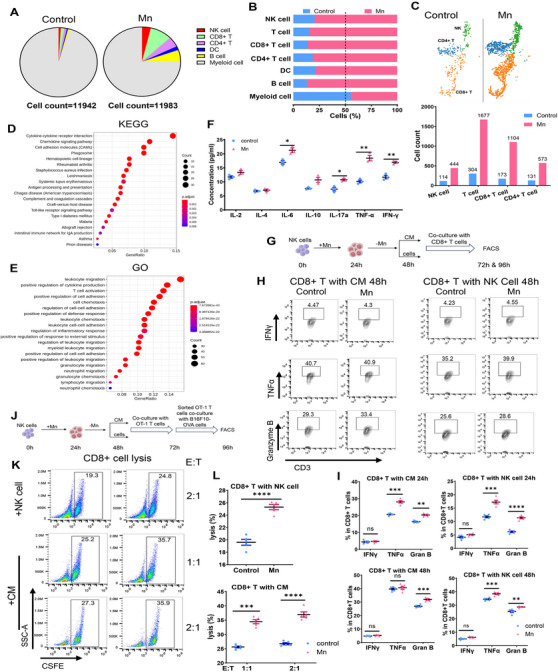
Mn^2+^ boosts the activation of CD8+ T cells by functioning on NK cells. (A–E) The B16F10 tumor models were established, as shown in Figure 1(A). The tumors were collected after three injections of Mn^2+^ for single‐cell sequencing analysis (*n* = 3 per group pool). The composition ratio (A), abundance difference (B), and quantity (C) of various tumor infiltrating immune cells were tested. KEGG (D) and GO (E) analysis were performed in NK cell cluster. (F) Primary murine NK cells were isolated from mice spleen and treated with MnCl_2_ for 24 h. The supernatant was collected for cytokine analysis by LEGENDplex immunoassay (*n* = 3 per group). (G–I) Mice CD8+ T cells were isolated from mice PBMCs and incubated with preactivated murine NK cells or supernatant (conditioned medium) (G). Representative FACS data (H) and statistics (I) of frequency of effector factors expressed by CD8+ T cells (*n* = 5 per group). (J–L) OT‐1 CD8+ T cells were incubated with preactivated murine NK cells, and then cocultured with OVA‐expression target cells (J). Representative FACS data (K) and quantification (L) of cytotoxicity of OT‐1 CD8+ T cells against target cell (*n* = 5 per group). Data represent analyses of the indicated *n* mice per group, means ± SEM. ^*^ *p* < 0.05; ^**^ *p* < 0.01; ^***^ *p* < 0.001; ^****^ *p* < 0.0001; ns, not significant, *p* > 0.05.

The aforementioned studies demonstrated a partial impairment in tumor rejection when Mn^2+^ was administered, namely in cases where T cells or NK cells were depleted (Figure 1F,I), indicating the direct and NK‐mediated impact of Mn^2+^ on T cells. In order to assess the significance of Mn^2+^‐induced NK cell activation on CD8+ T cell activation, we conducted a detailed analysis of the functional marker of CD8+ tumor‐infiltrating lymphocytes (TILs) using NK cell depletion. The activation of CD8+ TILs was somewhat diminished after administering Mn^2+^ to the NK‐depleted mice, but not to the same extent as in the control mice (Figures 1K and S4). Therefore, we postulated that Mn^2+^ enhances the activation of CD8+ T cells, to some extent, via acting on NK cells. Single‐cell RNA sequencing (scRNA‐seq) analysis was conducted on the CD45+ tumor infiltrating cells to investigate the alterations in NK and T cell following Mn^2+^ treatment. Following Mn^2+^ treatment, there was a notable concurrent rise in the infiltration of NK T and CD8+ cells, as shown by both the proportion of CD45 + cells and the absolute numbers (Figure 4A–C). The gene ontology (GO) and kyoto encyclopedia of genes and genomes (KEGG) analyses on NK cells revealed notable activation of chemokine pathways and leukocyte migratory signaling in the Mn^2+^‐treated tumor, which aligns with the cell phenotyping and functional investigations (Figure 4D,E). Furthermore, the induction of Mn^2+^ resulted in improvements in the release of various cytokines from primary murine NK cells in vitro, including a notable enhancement of IFNγ, which has the potential to stimulate the activation of CD8+ T cells (Figure 4F).

We further elucidated the effect of Mn^2+^‐induced activation of NK cells on the functionality of T cells. Purified murine NK cells were subjected to a 24‐h exposure to Mn^2+^, followed by a 24‐h period without Mn^2+^. Subsequently, the CD8+ T cells were cultured with either NK cells or the conditioned medium for 24 or 48 h (Figure 4G). The activation of CD8+ T cells was greatly enhanced in both conditions of Mn^2+^‐incubated NK cells, particularly when cocultured with NK cells (Figure 4H,I). We conducted an extensive examination of the impact of Mn^2+^‐induced NK cells on the tumor‐specific killing of CD8+ T cells using the coculture system (Figure 4J). The cytotoxicity of CD8+ T cells was dramatically boosted when managed with either Mn^2+^‐activated NK cells or conditioned medium (Figure 4K,L). In conclusion, these data suggest that Mn^2+^ exert a dual role in boosting CD8+ T cell activation, both through NK cell‐induced mechanisms and through direct effects.

### 2.4 Mn^2+^ activates NK cells through NK cell‐intrinsic cGAS–STING

**FIGURE 5:**
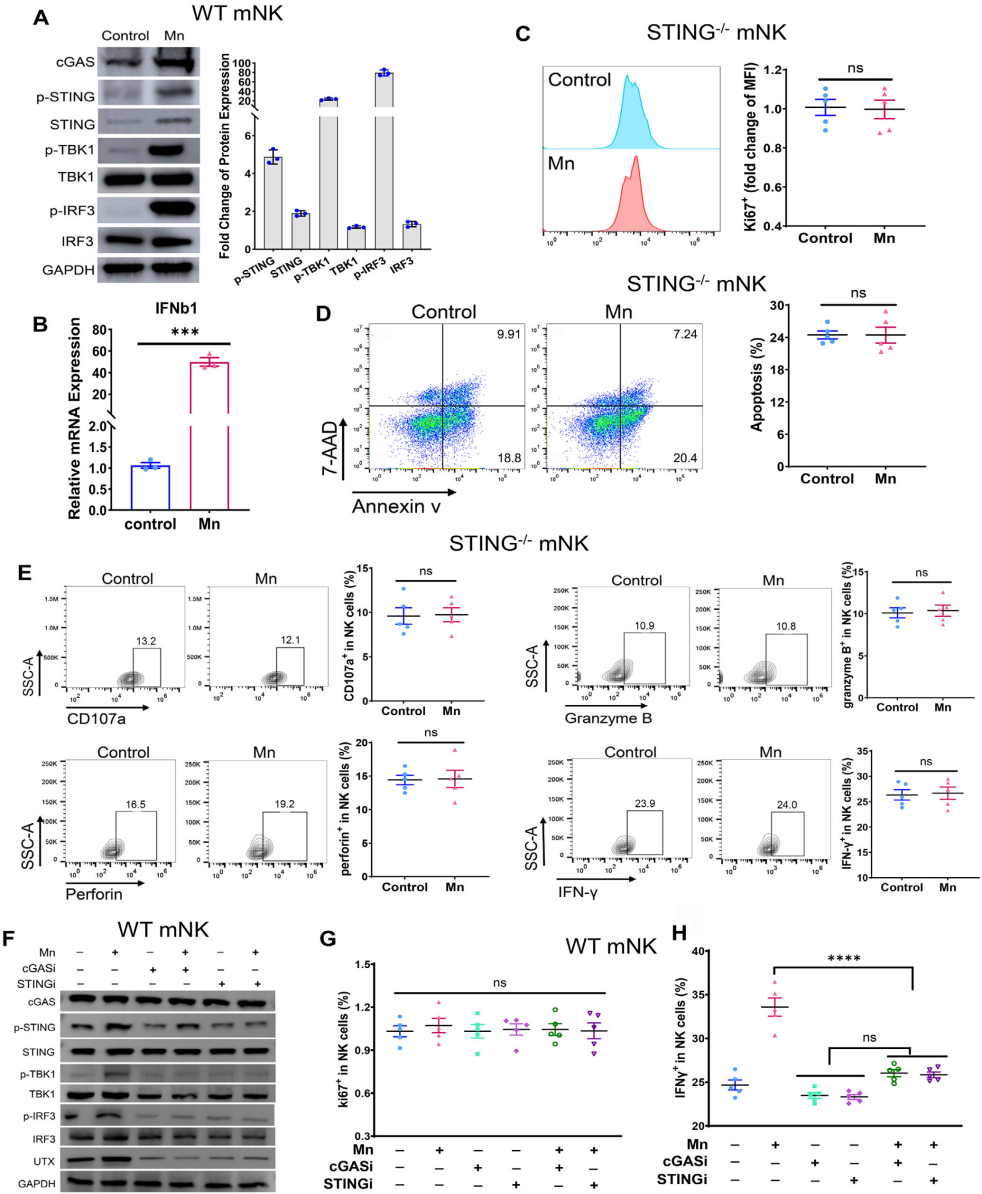
Mn^2+^ activates NK cells through NK cell‐intrinsic cGAS–STING. (A) Western blot of cGAS–STING signaling molecules in primary murine NK cells with or without Mn^2+^ treatment (*n* = 3 per group). (B) qRT‐PCR of IFNb1, the major effector indicator of cGAS–STING, in murine NK cells, in the presence or absence of Mn^2+^ (*n* = 5 per group). (C and D) Apoptosis (C) and proliferation (D) analysis of STING^−/−^ murine NK cells after Mn^2+^ treatment in vitro (*n* = 5 per group). (E) NK cells were isolated from STING^−/−^ mice and cultured as demonstrated in Figure 2A. Representative FACS data (left) and quantification (right) of effector factors of NK cells CD107a, IFNγ, granzyme B, and perforin (*n* = 5 per group). (F) Immunoblot analysis of cGAS–STING pathway in primary murine NK cells inhibited cGAS with RU.521 (cGAS inhibitor) or STING with H‐151 (STING inhibitor) during Mn^2+^ treatment in vitro. (G and H) Flow cytometry data of Ki67+ (G) and IFNγ+ (H) murine NK cells treated with Mn^2+^ and inhibitors of cGAS or STING in vitro (*n* = 5 per group). Data represent analyses of the indicated *n* mice per group, means ± SEM. ^***^ *p* < 0.001; ^****^ *p* < 0.0001; ns, not significant, *p* > 0.05.

In agreement with the established function of Mn^2+^ as a potent agonist of cGAS–STING, we detected a substantial increase in the phosphorylation of STING, TBK1, and IRF3 in primary murine NK cells after Mn^2+^ treatment. Meanwhile, there was a significant elevation in the expression of IFNb1 mRNA, which is a primary downstream indicator of STING activity (Figure 5A,B). Previous study has demonstrated that internal STING is necessary for the antitumor activity of NK cells. We subsequently concentrated on confirmation of the involvement of STING in the activation of NK cells triggered by Mn^2+^ utilizing the STING deficient (STING^−/−^) mice. The proliferation and apoptosis of murine STING^−/−^ NK cells were unaffected by the Mn^2+^ treatment, in accordance with NK cells from wild‐type WT mice (Figure 5C,D). Notably, the stimulation of NK cell activity by Mn^2+^ was completely abolished in the absence of STING in NK cells (Figure 5E).

Prior work has demonstrated that tumor‐specific cGAS can trigger STING signaling in NK cells via cGAMP transfer. Considering that Mn^2+^ can efficiently activate cGAS independent of dsDNA, we further explored whether Mn^2+^ enhances NK cell function by activating NK cell‐intrinsic cGAS. Inhibitor of cGAS (cGASi) exhibited minimal impact on STING pathway activity, but effectively eliminated the Mn^2+^‐induced elevation in phosphorylation of STING, TBK1, and IRF3, which are downstream targets of the cGAS signaling (Figure 5F). STING inhibitors have a comparable impact on STING signaling (Figure 5F). Although the proliferation of NK cells was not impacted by either cGASi or STINGi, the activation of NK cells, as indicated by the production of IFNγ, CD107a, and granzyme B, was substantially dampened by both inhibitors even in the presence of Mn^2+^ induction (Figures 5G,H and S5). Together, these results suggest that Mn^2+^ triggers the activation of the NK cell‐intrinsic cGAS–STING and subsequently boosts NK cell responsiveness.

### 2.5 Mn^2+^ induces UTX depending on STING to stimulate NK cell activation

**FIGURE 6:**
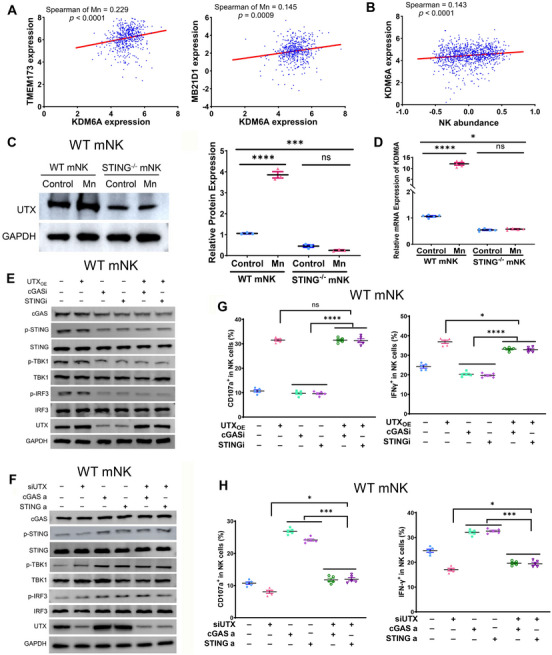
Mn^2+^ induces UTX expression depending on STING to stimulate NK cell activation. Spearman correlation between expression of KDM6A (UTX) and TMEM173 (STING, left) or MB21D1 (cGAS, right) employing the aforementioned scRNA‐seq data. (B) Correlation plot of KDM6A expression and abundance of tumor infiltrating NK cells in tumors with publicly available data from TCGA database. (C) Image of immunoblot (left) and quantification of expression of UTX (right) in primary NK cell from WT and STING^−/−^ mice, in the presence or absence of Mn^2+^ (*n* = 3 per group). (D) qRT‐PCR of KDMA6 in murine WT and STING^−/−^ NK cells with or without Mn^2+^ treatment (*n* = 5 per group). (E) Western blot of cGAS–STING pathway in primary murine NK cell with overexpression of UTX and treatment with inhibitors of cGAS or STING. (F) Immunoblot analysis of cGAS–STING pathway in primary murine NK cell with knockdown of UTX and treated with agonists of cGAS (G3‐YSD) or STING (2′3′‐cGAMP). (G and H) Flow cytometry data of CD107a+ (left) and IFNγ+ (right) NK cells with above mentioned (E and F) treatment (*n* = 5 per group). Data represent analyses of the indicated *n* mice per group, means ± SEM. ^*^ *p* < 0.05; ^***^ *p* < 0.001; ^****^ *p* < 0.0001; ns, not significant, *p* > 0.05.

Subsequently, we endeavored to get a more thorough comprehension of the molecular mechanism responsible for the activating effects of intrinsic cGAS–STING on NK cells. UTX has evidently played a critical role in the effector responses of NK cells.^39^ To determine the effect of Mn^2+^‐induced stimulation of cGAS–STING signaling on the expression of UTX, we assessed the relationship between cGAS–STING and UTX using our scRNA‐seq data. Significant correlations were observed between the transcript levels of MB21D1 (cGAS), TMEM173 (STING), and KDM6A (UTX) in the scRNA‐seq data of mice tumor infiltrating NK cells (Figure 6A). We conducted an exploration to identify the correlation between these genes using the publicly accessible data from the cancer genome atlas TCGA. The correlation between the expression of MB21D1, TMEM173, and KDM6A was once again verified using TCGA data, paralleling with our scRNA‐seq data (Figure S6A). Moreover, the quantity of KDM6A expression is directly associated with the abundance of NK cell infiltration in various types of cancers (Figure 6B). Notably, the presence of Mn^2+^ augmented the levels of both UTX mRNA and protein in primary murine NK cells in vitro (Figure 6C,D). However, this elevated expression is diminished in STING^−/−^ NK cells (Figure 6C,D). These data suggest that Mn^2+^ activates intrinsic cGAS–STING in NK cells to elevate UTX expression.

We further verified whether Mn^2+^‐induced intrinsic cGAS–STING activation on NK cell responsiveness was carried out by the expression of UTX. Overexpression or knockdown of UTX in primary mouse NK cells did not have a substantial impact on the downstream signaling of cGAS–STING, indicated as the phosphorylation of STING, TBK1, and IRF3. This was observed regardless of the treatment of cGAS or STING inhibitors or agonists (Figure 6E,F). Furthermore, it has been observed that the presence of cGAS or STING inhibitors does not impede the ability of UTX to greatly boost NK cell activation, as evident by the increased production of IFNγ, CD107, granzyme B, and perforin (Figures 6G and S6B). Likewise, the repression of UTX during the administration of cGAS or STING agonists resulted in the eradication of cGAS–STING‐induced activation of murine NK cells (Figures 6H and S6C). Collectively, these results indicated that Mn^2+^governs the expression of UTX by activating the cGAS–STING signaling, which subsequently boosts the responsiveness of NK cells.

## 3 DISCUSSION

As cytotoxic lymphocytes, NK cells can be a valuable complement to immunotherapies that modulate T‐cell responses, especially crucial for cancers without T‐cell‐specific antigens or major histocompatibility complex (MHC)‐class I molecules.^40^, ^41^, ^42^ The data indicate that STING signaling may play a vital role in regulating NK cell function.^43^ However, the underlying molecular mechanism still needs to be better understood. However, the unique characteristics of STING agonists pose multiple obstacles in their practical application. This study utilized Mn^2+^, a cGAS–STING agonist proven safe for clinical practice,^44^ to affect the direct cytotoxicity and cytokine secretion of NK cells in vitro, regardless of MHC expression. Furthermore, it augmented the antitumor efficacy of NK cells in vivo. Our findings offer a potentially promising option for immunotherapy that targets NK cells.

Early studies, which focused on observable characteristics, indicated the regulatory effect of Mn^2+^ on NK cells.^45^, ^46^ However, the underlying mechanism remains unclear. Mn^2+^ is an essential metal element in the human body that exerts function in multiple physiological functions by regulating various Mn‐dependent enzymes. Mn^2+^ simultaneously stimulates the immune response through different mechanisms, including nutritional immunity, The NLR family pyrin domain containing 3 (NLRP3) inflammasome, ataxia‐telangiectasia mutated (ATM)–p53 pathway, and cGAS–STING signaling.^47^, ^48^, ^49^ The exact process by which Mn^2+^ is involved in the regulation of NK cell has yet to be established. Our previous study indicates that Mn^2+^ could function as a cGAS–STING agonist, stimulating the augment of CD107a and Granzyme B in NK cells.^44^ However, further evidence is required to determine its regulatory effect and mechanism on NK cell activity. This work validated the cytotoxicity and responsiveness of NK cells, which were boosted by the activation of cGAS–STING induced by Mn^2+^. The experiments were conducted using STING^−/−^ mice and inhibitors of cGAS or STING. Furthermore, further study demonstrated that the cGAS–STING signaling plays a vital role in controlling the activity of NK cells by regulating the expression of UTX. This brings further insight into the impact of Mn^2+^ on the regulation of NK cells.

We observed a simultaneous enhancement of the killing ability and cytokine secretion of NK cells due to the addition of Mn^2+^. Antitumor immunity is the result of the orchestrated modulation of different cells within the tumor microenvironment.^50^, ^51^ It is crucial to investigate whether Mn^2+^ affects other immune cells by modifying NK cells’ secretory function to facilitate antitumor responses. The boosting effect of Mn^2+^ on tumor‐infiltrating T cells was significantly reduced in NK cell‐depleted mice, indicating the vital role of NK cells in regulating the effect of Mn^2+^ on T cells. In addition to the known direct effects that Mn^2+^ has on T cells,^44^ a coculture in vitro model also showed that Mn^2+^ has indirect effects on T cells by activating NK cells. Our discovery broadens our comprehension of the mechanism underlying the anticancer effects of Mn^2+^‐induced NK cells.

The cGAS–STING cascade activation can trigger the subsequent activation of the transcription factors IRF3 and type I IFN, resulting in the production of IFN‐stimulated genes, which include numerous cytokines and chemokines. Our data demonstrated that Mn^2+^ can stimulate the cGAS–STING signaling, leading to the increased production of various cytokines by NK cells, including TFNα, IFNγ, interleukin 6 (IL‐6), and IL‐17a. tumor necrosis factor‐alpha (TNFα) and IFNγ play a crucial role in the cytotoxicity of NK cells.^43^ Additionally, IFNγ has been indicated to contribute to the activation of CD8+ T cells.^52^ Meanwhile, despite being categorized as inhibitory cytokines, both IL‐6 and IL‐17a are pleiotropic cytokines.^53^, ^54^ They are able to stimulate CTL activation, which leads to the destruction of tumor cells. However, they also can attract suppressive immune cells like MDSCs to the tumor microenvironment, which hinders the function of CTLs.^55^ They primarily cooperate with other cytokines, such as TFNα and IFNγ, to delicately modulate the signaling in target cells according to the intracellular environment.

NK cell–intrinsic STING interacts with tumor cell‐inherent cGAS via cGAMP transport to enhance the antitumor response of NK cells.^30^, ^56^ NK cell‐based strategies, such as chimeric antigen receptor modified (CAR)‐NK or combination regimens, are regarded as practicable options for tumor immunotherapy.^57^, ^58^ A crucial requirement for clinical applications is to explore NK cell activation approaches in a tumor‐derived cGAMP‐independent manner through activating NK cell intrinsic cGAS. As a novel cGAS–STING agonist, Mn^2+^ can improve the sensitivity of cGAS to dsDNA and the binding capacity of STING to cGAMP.^44^ Significantly, Mn^2+^ can directly activate cGAS independent of dsDNA.^33^ The utilization of Mn^2+^ as an agonist can directly stimulate the inherent cGAS–STING cascade in NK cells, without relying on tumor cells, and enhances the responsiveness of NK cells.

The activation of the STING signaling has emerged as a promising approach for cancer therapy, and STING agonists have shown remarkable benefits in various preclinical tumor models.^59^, ^60^ Nevertheless, the utilization of STING agonists in trials is primarily restricted to intratumoral injection due to concerns over drug stability and toxicity.^61^ Consequently, their application is limited in scope and their efficacy is suboptimal. Given its widespread accessibility and well‐documented toxicity, Mn^2+^ has the potential to be a potent cGAS–STING agonist. The initial Phase I clinical trial has shown that when administrated systemically as a cGAS–STING agonist, Mn^2+^ is safe for clinical application and effective in multiple cancers.^44^ Mn^2+^ could function as a cGAS–STING agonist and contribute to innovative NK cell immunotherapies, such as CAR‐NK.

Our findings validate that Mn^2+^ can enhance the responsiveness of NK cells by activating NK cell–intrinsic cGAS and STING, independent of the existence of tumor cells. Furthermore, the activation of NK cells induced by cGAS–STING signaling is facilitated by the regulation of UTX expression, which offers valuable insights into cGAS–STING activation in NK cells. Furthermore, Mn^2+^ not only boosts the cytotoxicity of NK cells, but also modulates the production of cytokines by NK cells, thereby affecting the activity of T cells. This offers a logical explanation for the antitumor response of Mn^2+^. Mn^2+^, a potent cGAS–STING agonist, can be effectively utilized as a regimen for NK cell immunotherapy.

## 4 MATERIALS AND METHODS

### 4.1 Mice

Female C57BL/6J mice, aged 4−6 weeks old, were obtained from SPF (Beijing) Biotechnology Co. Ltd [License No. SCXK (Beijing) 2016‐0002]. The mice had a specific pathogen‐free (SPF) grade and weighed between 24 and 26 g. They were housed in groups of five mice per cage at a temperature of 24–26°C and a relative humidity of 60%. The mice were exposed to a 12‐h light‐dark cycle, and were provided with ample food and water. The animal experiment received approval from the Animal Ethics Committee of the Chinese PLA General Hospital. The rearing and experimental environments complied with the national regulations on animal experimentation, health inspection, and quarantine.

### 4.2 Cells and cell culture

B16F10 melanoma cells and K562 tumor cells, acquired from ATCC, were cultured in DMEM (Gibco) with the addition of 10% FBS (Gibco), 100 U/mL penicillin, and 100 mg/mL streptomycin. K562 cells additionally received 10 mM HEPES. The cells were incubated at 37°C in a 5% CO_2_ atmosphere. Both mouse and human primary NK cells were grown in DMEM with identical supplements, including 10 mM HEPES. The NK‐92MI cell line, obtained from ATCC, was cultured in MEM medium supplemented with 12.5% horse serum (Hyclone), 12.5% fetal bovine serum (Gibco), 0.02 mM folic acid (Sigma), 0.2 mM myo‐inositol (Sigma), 1% penicillin/streptomycin, and 0.1 mM b‐mercaptoethanol (Gibco). The absence of mycoplasma contamination was verified in all cell cultures.

### 4.3 Mouse primary NK cell isolation

Spleens were harvested from mice and placed on a 70‐mesh grinding net. Then, the spleens were gently grinned using a syringe piston with a small amount of PBS. Then, the diluent was slowly mixed with splenocytes to the lymphocyte isolation solution along the wall of the centrifugal tube and centrifuged at 2000 rpm for 25 min. Further, the middle layer of single nucleated cells was collected. The cells were washed once with PBS and then NK cells were sorted with the mouse NK cell isolation magnetic beads (Miltenyi Biotec; cat 130‐115‐818). Primary NK cells were cultured with the addition of IL‐2 and IL‐15.

### 4.4 Human primary NK cell isolation

PBMCs were isolated from peripheral blood samples of healthy volunteers. Written informed consent was obtained from all participants. The blood sample was mixed with an equal volume of PBS, then layered with Ficoll (Solarbio) and subjected to centrifugation at 2000 rpm for 20 min at a room temperature. PBMCs were collect from the middle layer of single nucleated cell and sorted with the human NK cell isolation magnetic beads (Miltenyi Biotec; cat 130‐092‐657). Primary marine NK cells were cultured with the addition of IL‐2 and IL‐15.

### 4.5 Mouse subcutaneous tumor models

Either 2 × 10^5^ or 1 × 10^5^ B16F10 cells were resuspended in 100 µL of PBS and then injected subcutaneously to the flanks of wild‐type or Rag1^−/−^ C57BL/6J mice, respectively. Treatment of 5 mg/kg MnCl_2_ was started 3 days after tumor cell injection, as shown in Figure 1A,E,H. In the NK cell‐depleted mice model, 400 µg of anti‐NK1.1 antibody (clone PK136; BioXCell) was administered intraperitoneally every 3 days, starting 1 day before the injection of tumor cells. Flow cytometry of PBMC was used to confirm the depletion of NK cells.

### 4.6 In vitro cellular analysis

Preactivated primary murine or human NK cells were or not exposed to 100 µM MnCl_2_ for 24 h. Subsequently, the activated cells were gathered and subjected to flow cytometry to assess the quantity of cytotoxic factors. In order to assess the effectiveness of tumor destruction, the NK cells from mice were cultured with K562 targets, whereas the NK cells from humans were exposed to either RMA‐S or RMA cells.

To evaluate T cell cytotoxicity, preactivated murine NK cells were cultivated for 24 h with or without 100 µM MnCl_2_, followed by an additional 24 h without Mn^2+^. Subsequently, the cells and supernatant (also known as conditioned medium) were collected separately. T cells were extracted from the spleen of mice and subjected to preactivation. Subsequently, they were cocultured either with NK cells or with conditioned medium. An analysis using flow cytometry was conducted to evaluate the cytotoxic factor and the ability to destroy tumor cells.

### 4.7 Flow cytometry

Samples of tumors and spleens were resected from mice at the time scheduled and mechanically smashed down to produce a single‐cell suspension. PBMCs were extracted from the peripheral blood of the mouse tumor models. Ex vivo cultured cells were harvested at the time specified. Immune cells were identified and functional parameters were evaluated using flow cytometry after staining the cells. Cells were fixed for intracellular labeling using cell nucleus fixation/permeabilization buffer (eBioscience). Data acquisition was conducted using a Beckman DxFLEX flow cytometer (Beckman COULTER) and processed using the Flowjo Software (BD Biosciences). All the antibodies used are listed in Table S1.

### 4.8 Quantitative RT‐PCR

The TRIzol Reagent (Thermo Fisher Scientific) was used to extract the total RNA. Reverse transcription was performed using the ReverTra Ace qPCR RT Master Mix (Toyobo) according to the recommended protocol. The qPCR analysis was conducted using the SYBR Green Real‐time PCR Master Mix (Toyobo) on the 7500 Fast Real‐Time PCR System with a 96‐Well Block (Applied Bio‐systems). The data were standardized based on the expression levels of GAPDH. The primer sequences are listed in Table S2.

### 4.9 Western blotting

The cells were rinsed with ice‐cold PBS twice, then subjected to lysis using RIPA buffer (Bioss) containing protease inhibitors. The protein concentrations of the total lysates were determined using the BCA assay and adjusted using the extraction reagent. The Western blot experiments were conducted using the antibodies specified in Table S1. Band intensity was assessed using the ImageJ software (NIH). By employing GAPDH staining, the loading sample was standardized.

### 4.10 NK cell cytokine in vitro analysis

Primary murine NK cells were cultured in vitro with or without 100 µM MnCl_2_ for 24 h. The supernatant was collected for the cytokine secretion assay using LEGENDplex flow cytometry‐based multiplex immunoassay (mouse anti‐virus response panel, 7406024; BioLegend). Data acquisition and analysis were performed on Beckman DxFLEX flow cytometer (Beckman COULTER) and LEGENDplex Data Analysis Software (BioLegend), respectively.

### 4.11 scRNA sequencing and data analyses

Tumors derived from mice, both treated with and without MnCl_2_, were harvested and enzymatically digested to obtain single‐cell suspensions. The 10× library preparation and sequencing were carried out in accordance with the standard procedure established by Shanghai Biotechnology Corporation. The cells were clustered together using graph‐based clustering of the PCA‐reduced data using the Louvain Method, after the calculation of a common closest neighbor graph. The identification of cell type was performed using SingleR and known marker genes. We performed differential expression analysis specifically on NK cells. Subsequently, we conducted GO and KEGG pathway enrichment analyses to identify the biological functions and signaling pathways associated with the genes that were elevated by Mn^2+^ treatment.

### 4.12 Statistical analysis

The data were reported as mean ± SEM. The statistical analyses were performed using GraphPad Prism 10.0. For data that follows a normal distribution, we used unpaired two‐tailed Student's *t*‐tests or one‐way ANOVA with Bonferroni's multiple comparisons. The analysis of non‐normally distributed data involved the use of Wilcoxon matched‐pairs signed rank tests for two paired groups or Kruskal‐Wallis tests for more than two groups. Tumor growth analysis using a repeated‐measures ANOVA. Correlations were performed by Spearman correlation test.

## AUTHOR CONTRIBUTIONS

*Conception and design*: Qian Mei and Weidong Han. *Development of methodology*: Qianyi Ming, Jiejie Liu, Zijian Lv, and Qian Mei. *Acquisition of data (provided animals, acquired and managed patients, provided facilities, etc.)*: Qian Ming, Jiejie Liu, Zijian Lv, Tiance Wang, Runjia Fan, Yan Zhang, and Meixia Chen. *Analysis and interpretation of data (e.g., statistical analysis, biostatistics, computational analysis)*: Qianyi Ming, Jiejie Liu, Zijian Lv, and Tiance Wang. *Writing, review, and/or revision of the manuscript*: Qianyi Ming and Qian Mei. *Administrative, technical, or material support (i.e., reporting or organizing data, constructing databases)*: Yingli Sun and Qian Mei. *Study supervision*: Yingli Sun, Weidong Han, and Qian Mei. All authors have read and approved the final manuscript.

## CONFLICT OF INTEREST STATEMENT

The authors confirm that this article content has no conflict of interest.

## ETHICS STATEMENT

The animal study underwent review and approval by the Animal Ethics Committee of the Chinese PLA General Hospital (A2020‐066‐01). Peripheral blood was obtained from the healthy volunteers after collecting signed informed consent and approval from the Institutional Review Board of the Chinese PLA General Hospital (S2018‐182‐01).

## Supporting information

Supporting Information

## Data Availability

The data that supports the conclusions can be obtained from the corresponding authors upon reasonable request via email (Qian Mei: meiqnn@hotmail.com). The scRNA sequence data have been deposited in National Genomics Data Center, China National Center for Bioinformation/ /Beijing Institute of Genomics, Chinese Academy of Sciences (GSA: CRA017689) (CNCB‐NGDC, https://ngdc.cncb.ac.cn/gsa/).
