# Supplementary material for: Manganese boosts natural killer cell function via cGAS–STING mediated UTX expression
Source: MedComm (2020). 2024 Aug 28;5(9):e683. doi: 10.1002/mco2.683 (PMC11351689; doi:10.1002/mco2.683)
Supplement: Supplementary file 1 — Supporting Information [file MCO2-5-e683-s001.docx]

# Manganese Boosts NK Cell Function via cGAS-STING mediated UTX expression

Qianyi Ming^1,#^, Jiejie Liu^1,#^, Zijian Lv^1,#^, Tiance Wang^1^, Runjia Fan^1^, Yan Zhang^1^, Meixia Chen^1^, Yingli Sun^3,*^, Weidong Han^1,2,*^ , Qian Mei^1,2,*^

^1^ Department of Bio-therapeutic, the First Medical Center, Chinese PLA General Hospital, Beijing 100853, P. R. China

^2^ Changping Laboratory, Yard 28, Science Park Road, Changping District, 102206 Beijing, China

^3^ Central Laboratory, National Cancer Center/National Clinical Research Center for Cancer/Cancer Hospital & Shenzhen Hospital, Chinese Academic of Medical Sciences and Peking Union Medical College, Shenzhen 518116, China

## Supplementary Tables

### Table S1. Antibody information

| **Name** | **Manufacturer** | **Cata. number** |
| --- | --- | --- |
| **FSCS** | | |
| Anti-mouse-CD45-BV510 | BioLegend | Cat# 103137 |
| Anti-mouse-CD3-PE/Dazzle 594 | BioLegend | Cat# 100245 |
| Anti-mouse-NK1.1-AF488 | BioLegend | Cat# 108717 |
| Anti-mouse-CD107a-BV421 | BioLegend | Cat# 121617 |
| Anti-mouse-Perforin-PB | BioLegend | Cat# 154311 |
| Anti-human/mouse-Granzyme B-AF700 | BioLegend | Cat# 372221 |
| Anti-mouse-IFN-γ-BV650 | BioLegend | Cat# 505831 |
| Anti-mouse-Ki-67-FITC | BioLegend | Cat# 652409 |
| Anti-human-CD45-BV510 | BioLegend | Cat# 368525 |
| Anti-human-CD3-PerCP/Cy5.5 | BioLegend | Cat# 317335 |
| Anti-human-CD56-PE/Cy7 | BioLegend | Cat# 304628 |
| Anti-human-CD107a-BV421 | BioLegend | Cat# 328625 |
| Anti-human-Perforin-AF647 | BioLegend | Cat# 308109 |
| Anti-human-IFN-γ-AF488 | BioLegend | Cat# 502517 |
| Anti-human-Ki-67-PE | BioLegend | Cat# 350503 |
| **Western** | | |
| GAPDH Mouse mAb | Cell Signaling Technology | Cat# 97166 |
| cGAS Rabbit mAb | Cell Signaling Technology | Cat# 31659 |
| Phospho-STING Rabbit mAb | Cell Signaling Technology | Cat# 72971 |
| STING Rabbit mAb | Cell Signaling Technology | Cat# 13647S |
| Phospho-TBK1/NAK Rabbit mAb | Cell Signaling Technology | Cat# 5483S |
| TBK1/NAK Rabbit mAb | Cell Signaling Technology | Cat# 38066S |
| Phospho-IRF-3 Rabbit mAb | Cell Signaling Technology | Cat# 37829S |
| IRF-3 Rabbit mAb | Cell Signaling Technology | Cat# 4302S |
| UTX antibody | proteintech | Cat# 23984-1-AP |
| UTX Rabbit mAb | Cell Signaling Technology | Cat# 33510S |

### Table S2. Antibody information

| **Primer name** | **Forward** | **Reverse** |
| --- | --- | --- |
| mGAPDH | TGTGTCCGTCGTGGATCTGA | TTGCTGTTGAAGTCGCAGGAG |
| mIFNb1 | ATGAACTCCACCAGCAGACAG | ACCACCATCCAGGCGTAGC |
| mKDM6a | CGGGCGGACAAAAGAAGAAC | CATAGACTTGCATCAGATCCTCC |

## Supplementary Figures


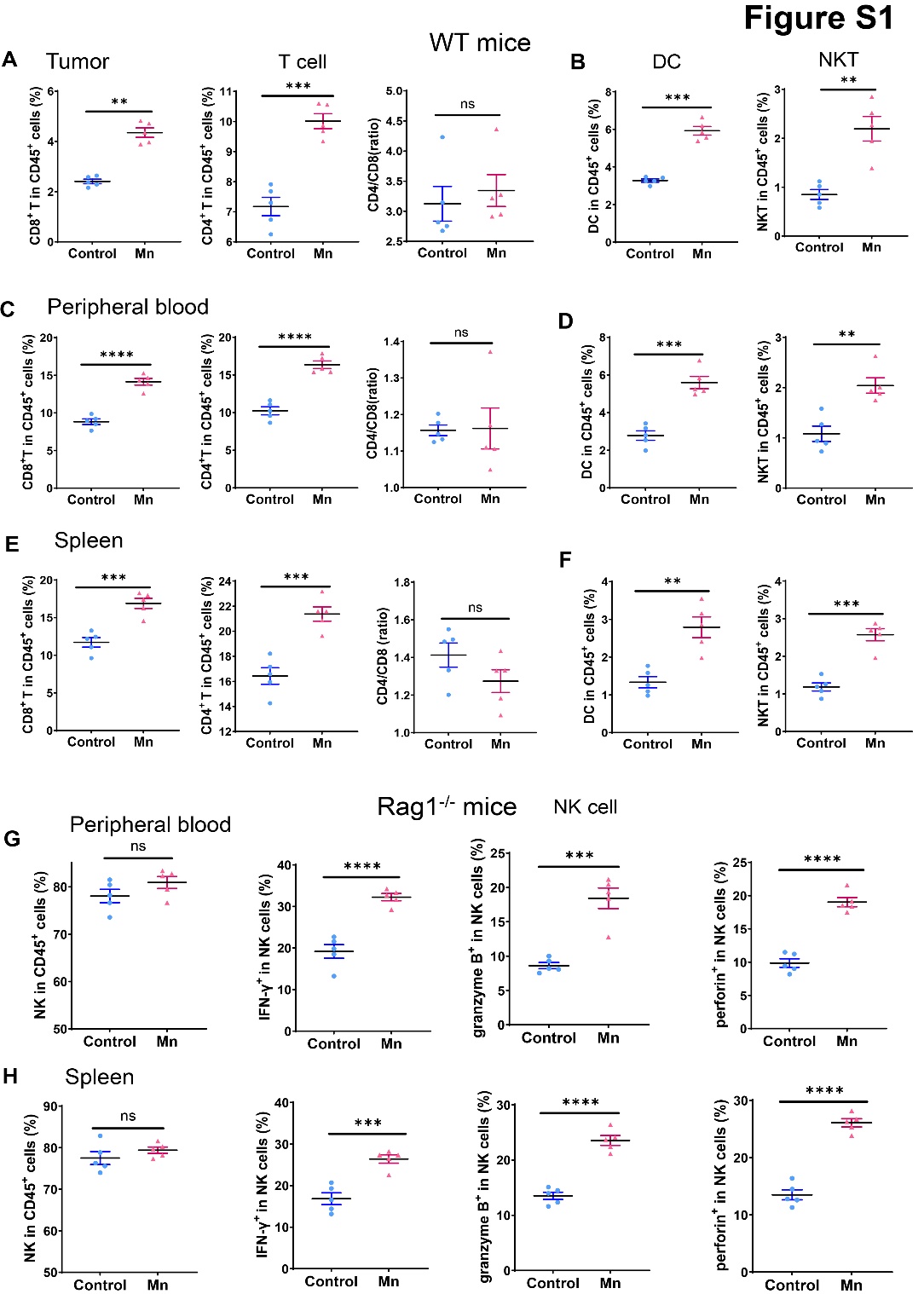


**Figure S1. Mn^2+^ promotes systemic antitumor immune responses.**

(A to F) The tumor models of WT mice were established and treated as in Figure 1. Flow cytometry data of T cells, DC and NKT cells from tumor inﬁltration (A, B), peripheral blood (C, D) and spleen (E, F) of WT mice. Frequencies of CD8+ and CD4+ T cell and ratios of CD4/CD8 T cells were presented in (A, C, E) and Frequencies of DC and NKT cells in (B, D, F) (n = 5 per group). (G, H) Tumors were inoculated into Rag1^-/-^ mice and treated with or without MnCl_2_. Flow cytometry data of quantity (left) and effector factor (right) of NK cells from peripheral blood (G) and spleen (H) (n = 5 per group). Data represent analyses of the indicated *n* mice per group, means ± SEM. ^**^ *P* < 0.01; ^***^ *P* < 0.001; ^****^ *P* < 0.0001; ns, not signiﬁcant, *P* >0.05.


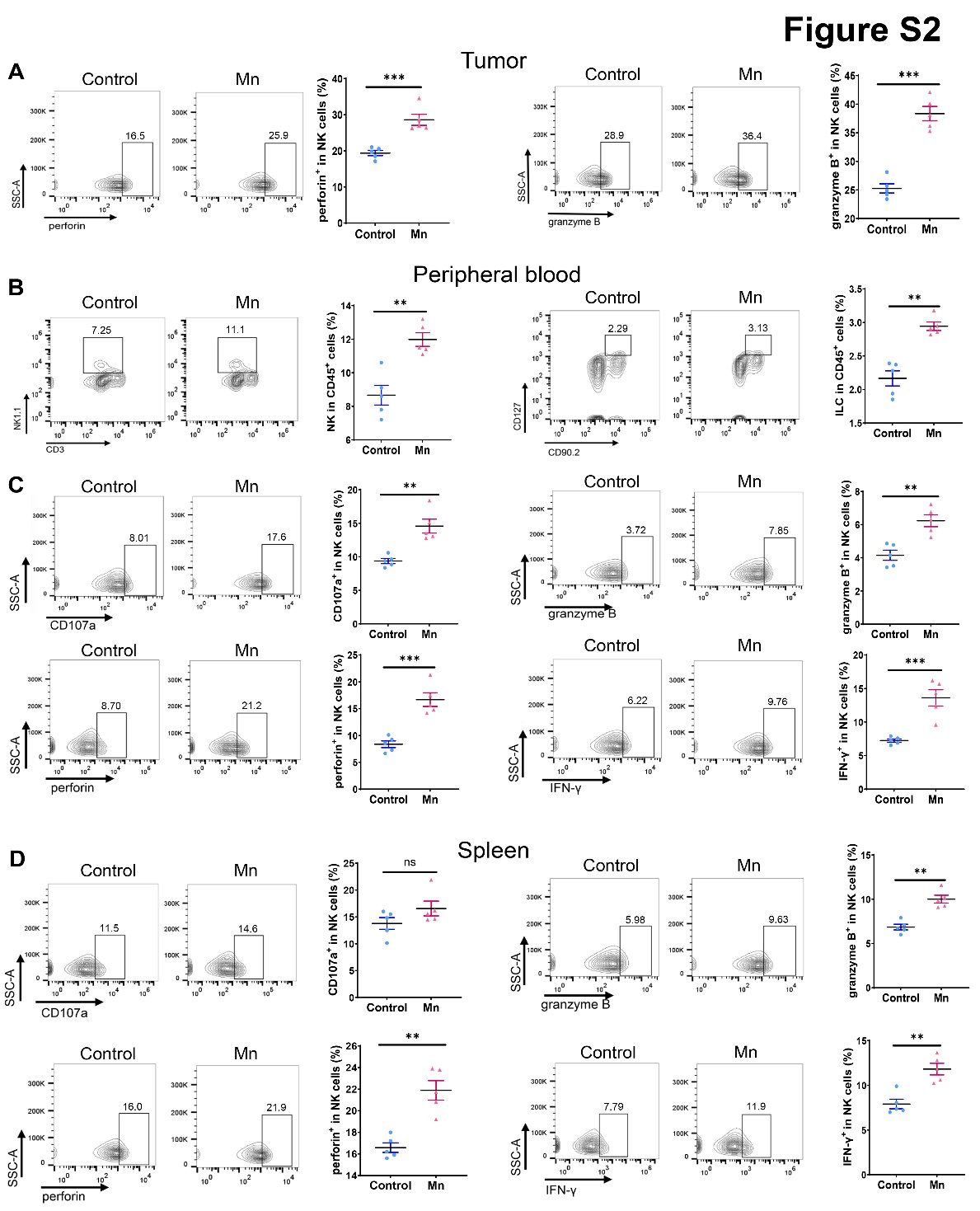


**Figure S2. Mn^2+^ stimulates systemic NK cell activation in WT and Tag1^-/-^ mice.**

(A) The tumor models of WT mice were established and treated as in Figure 1. Flow cytometry data and statistics of frequency of tumor infiltrating perforin+ and granzyme B+ NK cells (left) (n = 5 per group). (B to D) Flow cytometry data of NK cells, ILCs (B) and effector factors (C) of NK cells. (n = 5 per group). (D) Frequency of CD107a+, IFNγ+, perforin+ and granzyme B+ NK cells from mice spleen (n = 5 per group). Data represent analyses of the indicated *n* mice per group, means ± SEM. ^**^ *P* < 0.01; ^***^ *P* < 0.001; ns, not signiﬁcant, *P* >0.05.


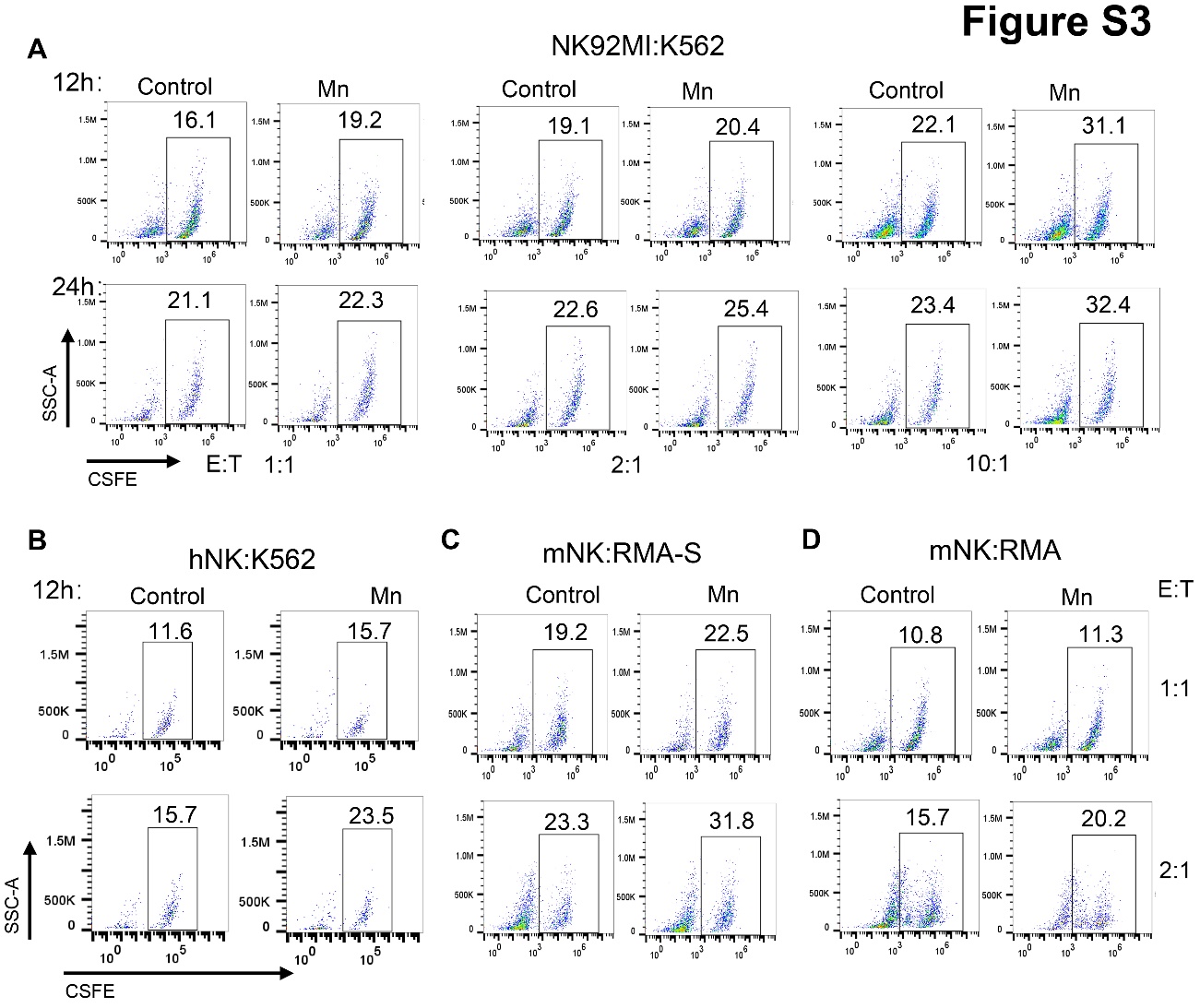


**Figure S3. Mn^2+^ boosts the cytotoxicity of NK cells against tumor cells.**

Representative FACS data of tumor killing ability of NK cells co-cultured with target tumor cells, as described in Figure 4(K).


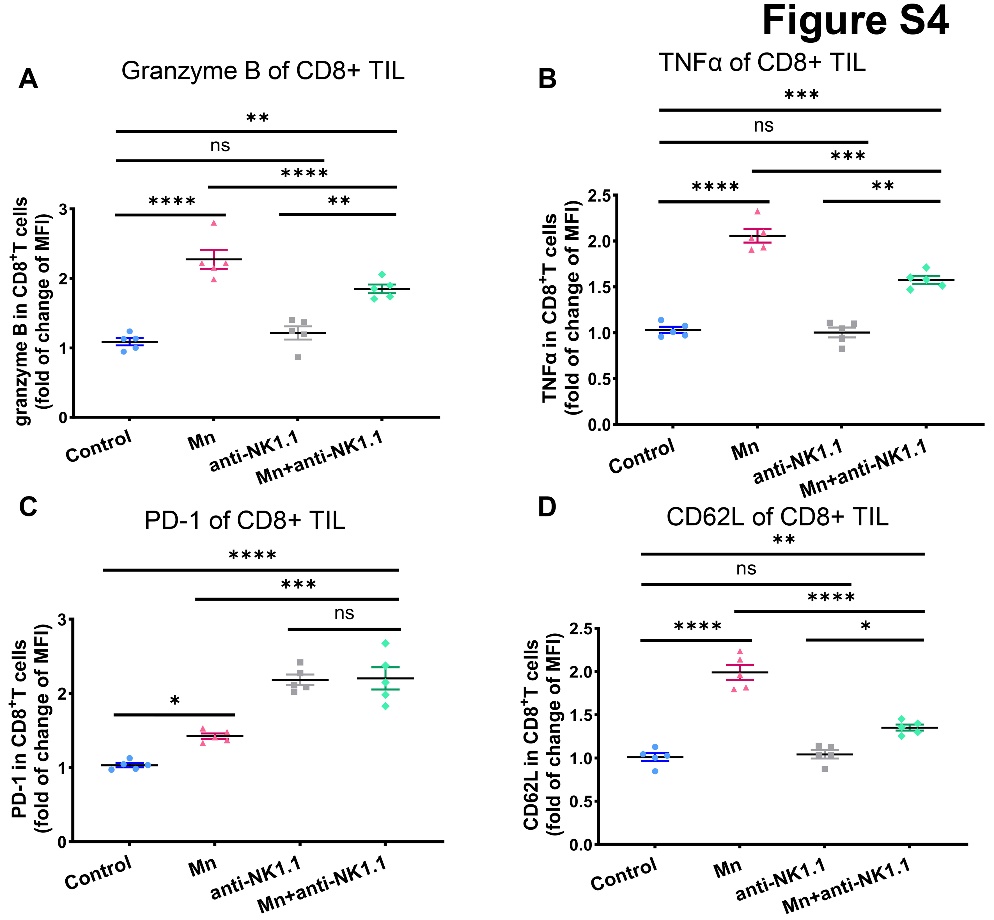


**Figure S4. Effect of Mn^2+^ on CD8+ T cells partially mediated by NK cells.**

Tumors were engrafted into the NK cell-depleted mice and administrated with or without MnCl_2_. Statistics of flow cytometry data of expression of granzyme B (A), TNFα (B), PD-1 (C) and CD62L (D) on CD8+ TILs (n = 5 per group). Data represent analyses of the indicated *n* mice per group, means ± SEM. ^*^ *P* < 0.05; ^**^ *P* < 0.01; ^***^ *P* < 0.001; ^****^ *P* < 0.0001; ns, not signiﬁcant, *P* >0.05.


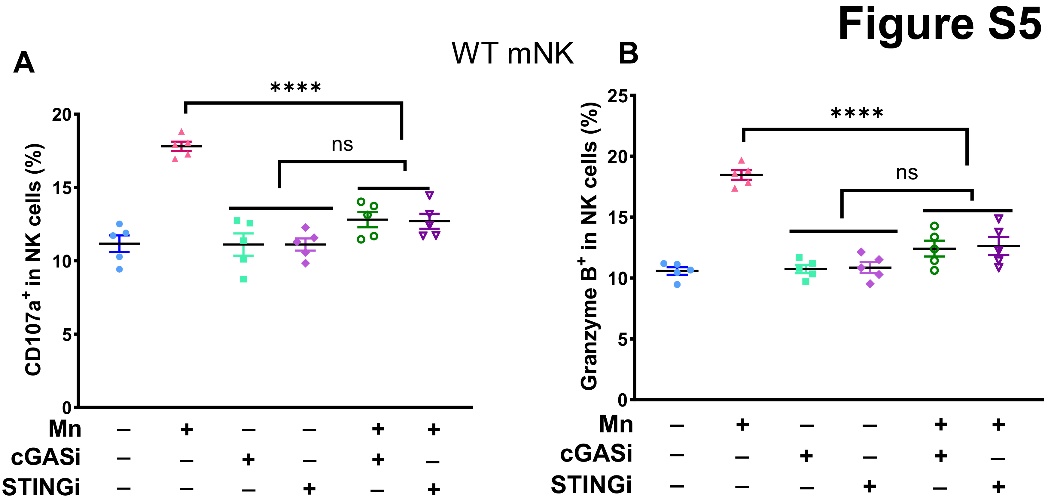


**Figure S5. NK cell-intrinsic cGAS-STING facilitates Mn^2+^-induced NK cell activation.**

Primary NK cells were isolated from WT mice spleen and incubated with Mn^2+^ and inhibitors of cGAS or STING, as indicated in Figure 5(F). Quantiﬁcation of frequency of tumor infiltrating CD107a+ (A) and granzyme B+ (B) NK cells (n = 5 per group). Data represent analyses of the indicated *n* mice per group, means ± SEM. ^*^ *P* < 0.05; ^**^ *P* < 0.01; ^***^ *P* < 0.001; ^****^ *P* < 0.0001; ns, not signiﬁcant, *P* >0.05.


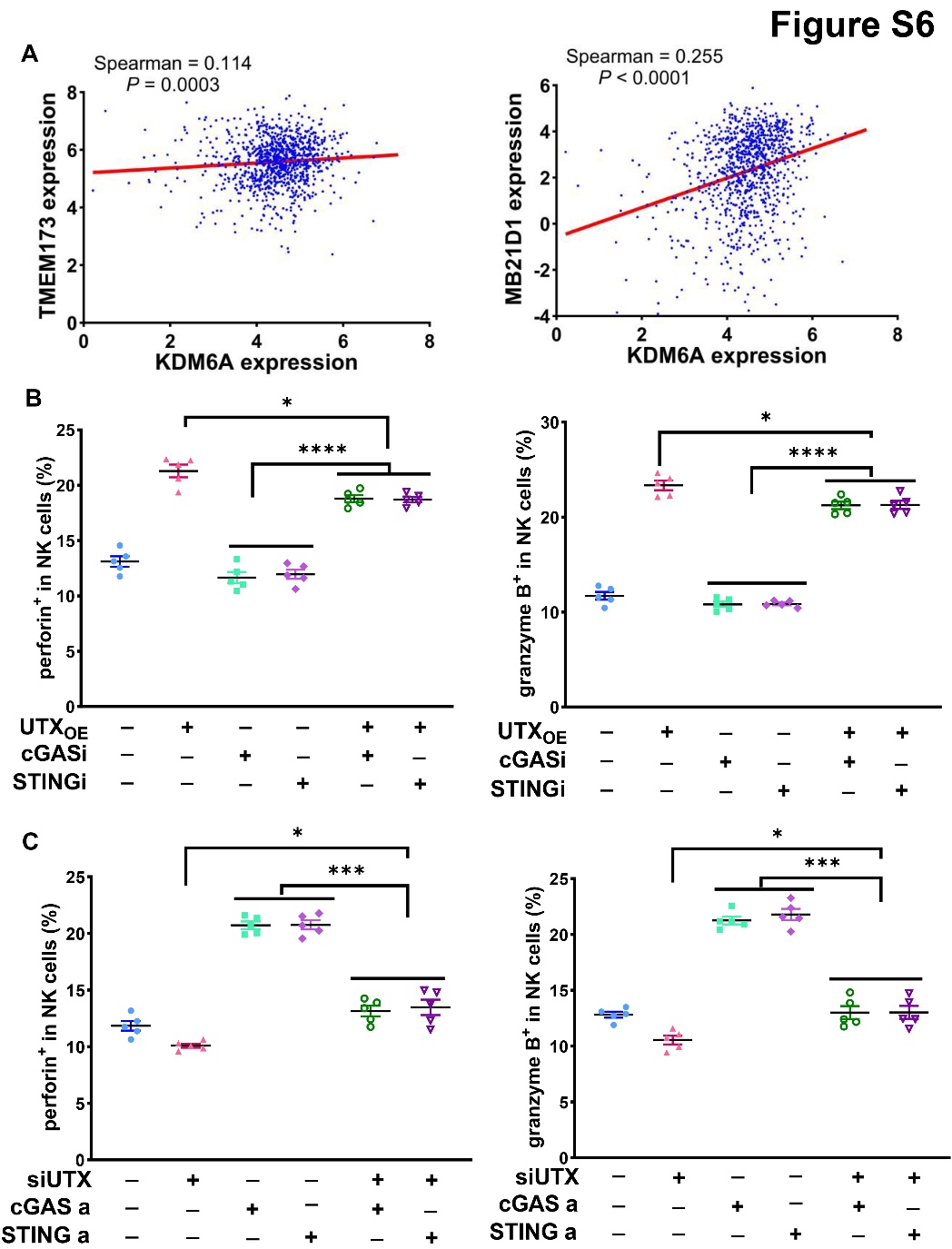


**Figure S6. Mn stimulates NK cell responsiveness depending on cGAS-STING-UTX.**

(A) Spearman correlation between expression of KDM6A (UTX) and TMEM173 (STING, left) or MB21D1 (cGAS, right) in tumors with publicly available data from TCGA database. (B, C) Primary murine mice NK cells were isolated from WT mice spleen and incubated as Figure 6(D, E). Quantiﬁcation of frequencies of perforin+ and granzyme B+ NK cells with overexpression (B) or knockdown of UTX (C) (n = 5 per group). Data represent analyses of the indicated *n* mice per group, means ± SEM. ^*^ *P* < 0.05; ^***^ *P* < 0.001; ^****^ *P* < 0.0001.
